# Decision aid use during post‐biopsy consultations for localized prostate cancer

**DOI:** 10.1111/hex.12613

**Published:** 2017-09-07

**Authors:** Margaret Holmes‐Rovner, Akshay Srikanth, Stephen G. Henry, Aisha Langford, David R. Rovner, Angela Fagerlin

**Affiliations:** ^1^ Centre for Ethics and Humanities in the Life Sciences Michigan State University East Lansing MI USA; ^2^ Department of Medicine Michigan State University East Lansing MI USA; ^3^ Henry Ford Hospital Wayne State University Detroit MI USA; ^4^ Division of General Medicine Geriatrics, and Bioethics University of California Davis Sacramento CI USA; ^5^ Department of Population Health New York University School of Medicine New York City NY USA; ^6^ VA Ann Arbor Centre for Clinical Management Research Department of Internal Medicine and Department of Psychology University of Michigan Ann Arbor MI USA; ^7^ Present address: Department of Population Health Sciences University of Utah Salt Lake City UT USA; ^8^ Present address: Salt Lake City VA Center for Informatics Decision Enhancement and Surveillance Salt Lake City UT USA

**Keywords:** decision aids, patient‐centred communication, prostate cancer, qualitative research, shared decision‐making, veterans

## Abstract

**Background:**

Decision Aids (DAs) effectively translate medical evidence for patients but are not routinely used in clinical practice. Little is known about how DAs are used during patient‐clinician encounters.

**Objective:**

To characterize the content and communicative function of high‐quality DAs during diagnostic clinic visits for prostate cancer.

**Participants:**

252 men newly diagnosed with localized prostate cancer who had received a DA, 45 treating physicians at 4 US Veterans Administration urology clinics.

**Methods:**

Qualitative analysis of transcribed audio recordings was used to inductively develop categories capturing content and function of all direct references to DAs (booklet talk). The presence or absence of any booklet talk per transcript was also calculated.

**Results:**

Booklet talk occurred in 55% of transcripts. Content focused on surgical procedures (36%); treatment choice (22%); and clarifying risk classification (17%). The most common function of booklet talk was patient corroboration of physicians’ explanations (42%), followed by either physician or patient acknowledgement that the patient had the booklet. Codes reflected the absence of DA use for shared decision‐making. In regression analysis, predictors of booklet talk were fewer years of patient education (*P* = .027) and more time in the encounter (*P* = .027). Patient race, DA type, time reading the DA, physician informing quality and physician age did not predict booklet talk.

**Conclusions:**

Results show that good decision aids, systematically provided to patients, appeared to function not to open up deliberations about how to balance benefits and harms of competing treatments, but rather to allow patients to ask narrow technical questions about recommended treatments.

## 1 INTRODUCTION

Patient decision aids (DAs) describing treatment options and risk/benefit trade‐offs among treatments have been successfully developed and tested over several decades, beginning, for early‐stage prostate cancer, in 1988.^1^, ^2^. While DAs are effective information translation tools, they are not routinely used in clinical practice.^3^ Systematic reviews of DA tools show they increase patient knowledge, increase patient clarity about their own values, decrease decisional conflict and increase patient interest in active roles in decision‐making. However, despite growing support for shared decision‐making in practice guidelines and continued development of new DAs, little is known about how patients and clinicians actually use DAs during clinical encounters.

DAs have been increasingly incorporated into communication and decision‐making interventions.^4^ DAs are usually developed for those conditions that are preference sensitive, meaning conditions with competing treatment or screening options that offer similar survival, with different side‐effect profiles. Initial treatment for clinically localized prostate cancer provides the classic example of a preference sensitive decision, as mortality is almost equivalent among surveillance (either active surveillance or watchful waiting), radiation therapy and prostatectomy.^5^, ^6^ Side effects of prostatectomy and radiation can include erectile dysfunction and bladder and bowel dysfunction, while surveillance requires follow‐up testing and may cause anxiety about living with cancer.^7^, ^8^, ^9^, ^10^

DAs have been implemented both in preparation for the clinical encounter (with and without patient coaching) and within the clinical encounter. The most recent update of the Cochrane Review of DAs found that of 105 studies, implementation in preparation for the clinical encounter occurred in 85% of included studies.^1^ Both implementation strategies improved knowledge and more accurate patient risk perceptions.

While previous studies have shown that DAs have potential to positively impact both patient informing and patient‐clinician interaction, little is known about the role that DAs play during the exchanges between patients and clinicians. The impact of DAs on the clinical encounter is assumed more often than examined. Of the 105 studies included in the most recent Cochrane review,^1^ 10 studied the effect on communication. Of those, the five studies that implemented the DA in preparation for the consultation all used self‐report measures of decision‐making.^11^, ^12^, ^13^, ^14^, ^15^ To our knowledge, no previous study has used data from direct observation of patient‐clinician communication (ie, data from transcripts or recordings of clinic visits) to identify how patients and clinicians actually use and discuss DAs during encounters. Analysis of transcripts or recordings (rather than reports based on patient or clinician recollection) is not subject to hindsight bias^16^ and is generally considered the most accurate method for assessing the content of communication during clinic visits.^17^, ^18^

In this study, we analysed visit transcripts to investigate the content and communicative function of direct references to DAs in patients’ post‐biopsy urology clinic encounters during which initial treatment decisions for clinically localized prostate cancer were made. We analysed transcripts for these visits to inductively develop categories capturing content and function of direct references to DAs (“booklet talk”). We also examined patient and clinician characteristics associated with the presence of a reference to a DA during the encounter. Understanding what content is discussed in direct reference to a DA, and how the DA functions in the encounter fills a knowledge gap about patient‐clinician communication following standardized DA provision. Understanding how DAs are discussed during encounters can help researchers and clinicians to design more effective DAs and implementation strategies.

## 2 METHODS

Audio recordings and survey data were obtained from a multisite clinical trial that compared two prostate cancer DAs, to determine their relative impact on treatment choice. Patients undergoing prostate biopsies were recruited from four US Veterans Administration (VA) Health Systems (Ann Arbor, Durham, Pittsburgh and San Francisco) between September 2008 and May 2012. At recruitment, when the biopsy was performed, each patient was randomized to receive either a plain language DA (designed by the Michigan Cancer Consortium (MCC)^19^, or a standard language DA (designed by the National Comprehensive Cancer Network (NCCN) and American Cancer Society (ACS). The MCC DA was developed to use plain language and to adhere to the standards of the International Patient Decision Aids Consortium (IPDAS).^20^ The current version of the MCC DA can be found at www.prostatecancerdecision.org. The NCCN DA was chosen because of its high‐quality information and the high credibility of the sponsoring organizations. The current version of the NCCN DA can be found at https://www.nccn.org/patients/guidelines/prostate. Both decision aids used the terminology “watchful waiting” because the term, “active surveillance” was not a commonly used term when this study was conducted. Therefore, we use watchful waiting throughout. (More detailed quality analysis of the DAs can be found in the Appendix S1).

Block randomization was used to ensure that equal numbers of African American and low‐literacy patients received each decision aid. Physicians were aware that patients received a DA, but not given any further instructions in DA use. In addition to transcripts of audio recordings, survey data describing patient characteristics and self‐reported DA use were available for analysis from the parent trial.

Patients with clinically localized prostate cancer (Gleason score 6 or 7, PSA < 20 ng/mL) were asked to participate in audio recording of the first post‐biopsy encounter, the one at which the patient first received his diagnosis and discussed initial treatment options. Surveys were administered at three time points: biopsy, immediately before the physician encounter and 7‐10 days following the physician encounter. Patients were called 2 days before the physician encounter and reminded to read the DA, but were not informed of their diagnosis. They learned their diagnosis from the physician, with the exception of one site that followed a practice of giving the diagnosis over the telephone. Participants at that site were interviewed before the diagnosis phone call. Physician participants were urology residents and attending physicians. All provided demographic data at the time of recruitment. The study was approved by the VA Institutional Review Boards at each participating site; written informed consent was obtained from each patient and physician participant. The funding agencies had no role in conduct or reporting of the parent study or the analysis presented in this manuscript.

### 2.1 Measures from the parent study

We obtained descriptive data from the parent study. Survey measures completed by patients before the clinical encounter included patient literacy^21^ and numeracy^22^, preference for shared decision‐making^23^, prostate cancer treatment knowledge related to survival benefit and side‐effects associated with treatments,^24^, ^25^, ^26^ treatment preference, use of and satisfaction with DA and demographics (patients’ race, ethnicity, age, marital status and education).

A measure of the quality of physician informing was obtained through a transcript analysis. The Informed Decision Making (IDM) score,^27^ is a standardized observational measure of the quality of physician informing behaviour, scored by analysing transcripts of audio‐recorded patient encounters.^28^ Patients’ PSA level, Gleason Score and treatment received were obtained from electronic medical records.

### 2.2 Audio recordings and transcripts for this analysis

A research associate sets up an audio recorder in the examination room at the start of each visit and then waited outside the examination room until the visit was complete. Recordings were later anonymized and transcribed verbatim. Of 256 transcripts, 252 were available for inclusion. Two transcripts were excluded because of recorder malfunction; two encounters were only to obtain a referral to radiation oncology. Time in the encounter was measured directly from the audio recordings. Time when the physician was out of the room was subtracted from total time to yield the net time the physician was in the room with the patient.

### 2.3 Coding and qualitative analysis

In this analysis, we coded and analysed direct references to the DA and used a two‐step coding process to identify the content categories and function categories to describe how the DA functioned in the exchange. In step one, two coders independently identified all direct DA references. In addition, a word search of the text was performed using the words “booklet”, “pamphlet”, “book” and “decision aid” to check for missing episodes. Booklet talk was classified into one of four transactional categories: (i) patient initiates and doctor responds, (ii) patient initiates and doctor fails to respond, (iii) doctor initiates and patient responds and (iv) doctor initiates and patient fails to respond. Coding exchanges (ie, topic initiation and response) accounts for the interactional nature of clinic visits and is a common approach when coding patient‐clinician communication.^29^, ^30^

Because we previously noted that communication tasks during these visits occurred in a predictable sequence,^31^ we analysed a random sample of 28 transcripts to evaluate whether booklet talk also occurred in predictable portions of visits, for example. after diagnosis delivery, during treatment choice discussions, at the close of the encounter. To do this, we calculated the percentage of total words in each transcript before each episode of booklet talk and analysed the distribution of results in the 28 randomly sampled transcripts. The wide distribution of percentages of words before episodes of booklet talk (range = 1‐99) and no clear clustering pattern, suggested there was no part of the clinical routine that triggered booklet talk. We therefore did not pursue a separate structural analysis of the encounters.

In step two, we inductively developed the set of content and function codes for each coded exchange by carefully analysing a random pilot set of fourteen transcripts. No constraints were placed on identification of content. Seven investigators independently applied the initial coding system to a second set of fourteen transcripts, resolved disagreements and modified the coding system until the codes could be applied reliably. Resulting content codes were: (i) treatment options, (ii) side‐effects, (iii) treatment choice/decision, (iv) risk classification, (v) nature of cancer and (vi) booklet quality.

Function codes captured the conversational work being done by booklet talk during the exchanges. Resulting function codes identified that the speaker: (i) acknowledges the booklet, (ii) gives advice or information, (iii) confirms or validates what was said, (iv) flags record‐keeping opportunity, (v) requests information, (vi) uses booklet to question doctor and (vii) expresses concern or fear. Complete definitions can be found in Tables 1 and 2. Each instance of booklet talk had at least one content and one function code. Codes were not mutually exclusive, (eg, an exchange could have more than one content and/or function code). We found no booklet talk exchanges that could be considered shared decision‐making. A final set of coding rules with examples was developed. Complete coding rules are available from the corresponding author.

**Table 1:** Booklet talk content codes

Content code	Content code definition	Frequency (%)
Treatment options	Booklet is referenced when discussing different treatment options.	36
Treatment choice/decision	Booklet is referenced when discussing making the actual treatment decision	22
Risk classification	Booklet is referenced when discussing PSA, grade and stage.	18
Side‐effects	Booklet is referenced with respect to side‐effects of treatment options	8
Nature of cancer	Booklet is referenced with respect to the generally slow growth of early‐stage prostate CAs	8
Booklet quality	Any positive or negative statements regarding the quality or utility of the booklet	8

**Table 2:** Booklet talk function codes

Function code	Function code definition	Frequency (%)
Learn more or confirm/validate	Doctor or patient utilizes the booklet to learn more or validate something read in the booklet	41
Acknowledges booklet	Doctor or patient acknowledges the patient has the booklet	28
Request for information or question	Patient utilizes the booklet to ask a question	12
Advice or information giving	Doctor utilizes the booklet to give advice or provide the patient with more information	6
Record‐keeping	Doctor or patient suggests writing notes in the booklet	5
Uses booklet to question doctor	Patient uses the booklet to challenge the physician	4
Expression of concern	Doctor or patient expresses concern specifically from something seen in the booklet	4

Six coders working in 3 pairs applied the final coding system to all 252 transcripts. Two coders independently coded each transcript; discrepancies were resolved by consensus. Coders were blinded to plain language vs standard language DA allocation. For rates of the presence or absence of booklet talk, the unit of analysis was the transcript. For frequency of occurrence of content and function codes and for frequency of speaker exchanges, the unit of analysis was the total number of coded exchanges. To describe the distribution of content codes across all transcripts, we compiled all instances of each content code that appeared in the codebook. The denominator for this analysis (298 codes) exceeds the number of episodes across all transcripts because an episode could include more than one topic. Coding was completed in Dedoose.^32^ Dedoose is, a Rich Internet Application (RIA) that allows data analysis and handling from mixed methods research. Frequencies and descriptive statistics were calculated using Microsoft Excel.^33^

### 2.4 Regression analysis

To identify predictors of booklet talk during the consultation, we conducted two mixed effects logistic regression models, using patient, physician and encounter level variables from the parent study to predict the presence of booklet talk in the transcript. Among the variables available in the parent study, we prioritized those with a theoretical relationship to the likelihood of mentioning the DA in the encounter. Patient education and race have been previously associated with how much patients participate in encounters with physicians.^34^, ^35^, ^36^, ^37^ Time spent reading the DA before the encounter was included as a measure of interest in the content. Age was not included because of the narrow range of patient ages. Time in the encounter, measured in minutes from the recordings, was included because trials of DAs have been shown across studies to sometimes shorten and sometimes lengthen encounter times.^38^, ^39^ DA type (plain vs standard language) was also included as a variable, as randomization in the original study was based on DA type.

The binary booklet talk variable (yes/no) was used as the main outcome in the logistic regression models. Specifically, to account for patient, visit and physician variables in the models, two mixed effect logistic regression models were conducted in Stata data analysis and statistical software version 14.0^40^ (using the *melogit* command). The first model only included patient and visit level variables (ie, education, race, DA type, time spent reading the DA and time in the encounter), with physician ID number treated as a random intercept to account for potential variation by physician. The second model added physician level variables, including physician age and the IDM score. For completeness, we only evaluated cases that had complete data for each variable in the models, making the final n in the mixed effects model 236/252 transcripts.

## 3 RESULTS

Demographic characteristics for the 252 patients are shown in Table 3. The mean age of the patient sample was 63.3 years (SD = 5.9); 33% were non‐white; 40% had high school education or less. The mean age of 45 treating physicians was 33 (SD = 7.2); 20% were women, 34% were non‐white. On average, each physician was recorded in 6 clinical encounters (SD = 4.3) and was 10 years post‐graduation.

**Table 3:** Participant characteristics

	Sample (n = 252)
Age	M = 63, SD = 6.01
Race (%)	
Caucasian	185 (73)
African American	67 (27)
Other	0 (0)
Education (%)	
<High school	5 (2)
High school grade/trade	79 (31)
Some college/Assoc.	116 (46)
BA+	52 (21)
Marital status (%)	
Married/partner	131 (52)
Divorced/separated	94 (37)
Widowed	7 (3)
Single	20 (8)

References to a DA, (“booklet talk”), occurred in 138/252 transcripts (55%). In the 138 transcripts containing booklet talk, there were 214 separate booklet talk episodes, with a maximum of 5 in a single transcript, a mean of 1.55 (SD .81) and a mode of one. Of the 214 booklet talk episodes, 120 (56%) were patient initiated. The observed rate of booklet talk per transcript was consistent with the rates reported in the surveys. In the post‐encounter surveys, 55% of patients reported bringing the DA to the encounter, while 90% reported reading the DA before the encounter (data available on request).

### 3.1 Content and function

DAs were referenced most frequently during discussion of treatment options (36%). The most common specific content code was details of surgery. Direct references to making a treatment decision constituted 22% of all content codes; clarification of technical information about risk classification, 18%. Frequencies for all content codes are shown in Table 1, and examples of each content code appear in Table 4.

**Table 4:** Examples of booklet talk content codes

**Treatment options**
*Example 1*
PAT‐ Yeah, so removing the prostate effects what other body functions or anything?
DOC‐ That's basically it, urinary and erectile functions
PAT‐Ok
DOC‐ No other real body functions
PAT‐ Body doesn't need that
DOC‐ It needs it if you want kids, it needs it if you um. Yeah your body doesn't really need it
PAT‐ After reading that book, the radiation seems the better…… but that's not what you're saying, it's not really.
DOC‐ There's benefits and risks to both
*Example 2*
PAT‐ In the book there was two types of radiation
DOC‐ Right there's the seeds that they can put into your prostate or radiation from the outside, where they focus all the energy
PAT‐ Some beam or something
DOC‐ Yeah external beam radiation
**Treatment choice/decision**
*Example 1*
PAT‐ I'll just come back in like two to four weeks.
DOC‐ Okay, alright. Um, I think that's a very, very reasonable, um again, this is a low risk prostate cancer. You've got good treatment options available, and um, you'll have the reading material that you got from to kind of help you navigate these decisions. Um, if you have any other questions or concerns, don't hesitate to call back over here to the clinic, you can talk to whichever one of the doctors is down here.
PAT‐ Okay.
DOC‐ Okay, and um, you know, we're happy to kind of help you make whatever decision it is that you want to make, whether that's surgery or radiation.
*Example 2*
DOC‐ Okay um, and even with aggressive disease….. the chance it can affect your lifespan at five years is low. It interests me that some patients say, “Listen I really want this tumor out.” And we get the tumor out, it's cancer, even despite the fact that I tell them that not all cancer is the same.
PAT‐ Sure
DOC‐ Okay
PAT‐ Well, like she had breast cancer and she immediately wanted it out. I basically said the same thing the other day. If I find out I have cancer I immediately want it out. But now, that I you know, read some of that and after talking with you I got a little, “Yeah it's, we'll do the wait and see approach for awhile.”
DOC‐ The only caveat about the wait and see approach again is that, you're a little different than the typical wait and see approach patient.
**Risk classification**
PAT‐ I'm, I'm confused about the three plus three, I have to interrupt you I'm sorry.
DOC‐ That's no problem you can feel free to interrupt as, ask me
PAT‐ This book is talking about a PSA number and then they're talking about a Gleason s….
DOC‐ Correct
PAT‐ What is the PSA number
DOC‐ His PSA is four point two
PAT‐ Four point two?
DOC‐ Correct
PAT‐ The, the, the Gleason number you're giving me you keep saying three plus three?
DOC‐ Or six
PAT‐ So his Gleason number is six?
DOC‐ Correct
PAT‐ In this book it's saying a Gleason number of six is not the slowest growing, it's the medium
DOC‐ No, it's the slowest growing.
**Side effects**
*Example 1*
DOC‐ Okay, what would you like to hear more about? I mean I guess I can talk most about the prostatectomy; do you have a thought?
PAT‐ Well, you know they have these questions and I might ask them in you know, how does the regular side effects in this booklet compare to the regular side effects in your practice.
DOC: Oh, and I'm not sure what's in the booklet, I should probably read this a little more closely.
*Example 2*
PAT‐ And what's that to do with, going the bathroom?
DOC‐ That is how you keep your urine in and not let it leak out
PAT‐ I thought I'd read some of that in there. I says, “I'd hate to have to run into a problem like that.”
DOC‐ Yeah. And that is a possibility, it is a slight possibility with radiation as well but not as much. But those are your biggest problems that we have, that we see with patients after surgery.
**Nature of cancer**
DOC‐ Yeah, and now we know so um we'll have you come back in a couple months
PAT‐ Ok
DOC‐ Alright
PAT‐ This type of cancer from what I read in the book is extremely slow growing
DOC‐ Yes, but you seem healthy enough that you will probably live another 20‐30 y at least
PAT‐ That's what I figured, you know I'm not that old yet
**Booklet quality**
DOC‐ Do you have access to the web?
PAT‐ Yep
DOC‐ It does a pretty good job of um, um it does a pretty good job about um, explaining treatment options and everything.
PAT‐ Okay, better than this book that I got?
DOC‐ Yeah
PAT‐ Really?
DOC‐ Yeah
PAT‐ That was pretty straight forward and simple and
DOC‐ The problem with those books is sometimes they are little bit out of date.

Function codes describe how the DA references were used in the encounter. As in the content codes, we compiled all instances of each function code that appeared in the codebook. The denominator for this analysis (316 codes), like content, exceeds the number of episodes. All exchanges were coded for both content and function. The functions were dominated by “learn more or validate” (41%) and “acknowledging the booklet” (28%). “Learn more” was usually a patient request to hear the physician's explanation for or interpretation of something the patient read in the DA. “Acknowledging the booklet” was usually a physician question about whether the patient received a DA or a comment that s/he saw the patient carrying a copy of the DA. The third most frequent category was consistent with the design of DAs, “using the booklet to ask a question” (12%). Examples of each category of function codes appear in Table 5.

**Table 5:** Examples of booklet talk function codes

**Acknowledges booklet**
*Example 1*
DOC‐ It gives you time to digest, you seem like the type of guy that you in good health will live a while longer so I do recommend some type of treatment but what you choose is up to you. Both are equally good
PAT‐ Is there literature
DOC‐ Yeah, they give you anything?
PAT‐ Yeah…..gave me one book
*Example 2*
DOC‐ And have you done some reading about it?
PAT‐ Yeah she gave me that book and that's the only thing and I'm not afraid of the procedure, it's just the
DOC‐ The possibility of being incontinent afterwards
PAT‐ Yeah
**Advice or information giving**
*Example 1*
DOC‐ You just have to remember like when you're coming to see urology. Okay, so, well I think if you all don't have any other questions, of course if you do have questions between now and then
PAT‐ Um‐hmm
DOC‐ Just feel free to call us, or if you change your mind, you read something in there and you say you know what I do want to talk to the radiation doctors just give us a call, we can set that up.
*Example 2*
DOC‐ Okay? So let's do this let's return to clinic in 3 wk. Alright does that sound like a good plan?
PAT‐ Yeah
DOC‐ And if you have any questions like I said, the handout is pretty good it's pretty detailed but it definitely will a
PAT‐ Yeah
DOC‐ You know help you maybe think through things and then talking to the oncologist or to the radiation doctors would be great
**Learn more or confirm/validate**
*Example 1*
DOC‐ um, for people that have high risk cancer and sometimes people have intermediate cancer we do get them the CAT scan and the bone scan to make sure it's nowhere else, but that's typically for higher risk, higher risk disease.
PAT‐ Yeah I was thinking, I read about that, that bone scan, CT scan and whatever other scans they've got. You wouldn't do that? I mean that's not, that's not an option to do?
DOC‐ Oh, it's, it's, it's typically you know, it's, it's not usually that it's not an option, it's always an option. It's just that for people with like low and intermediate risk prostate cancer, it's usually not necessary because the odds that it's spread are so low.
*Example 2*
PAT‐ Right, and can you put some information in my booklet?
DOC‐ I sure can, I can do that. Why don't I do this when we get finished with this because yeah, I'll put some information in here. What are your thoughts about what you've read here?
PAT‐ Well uh, I kind of thought that if it was low grade or anything, that uh, you know, we'd probably keep pretty close watch of it and uh, monitor it closely and so on and if it reaches a stage where, where, you know, where we determine that it needs pretty much, you know, prompt attention and so on and so forth, why we'll go ahead and give it to it, you know, you know, just go ahead and give it, do what's necessary then in that case. I was leaning a little bit towards treatment options of uh, now as far as percentage of cure and so on, radiation as compared to surgery, what are the basic percentages?
**Record‐keeping**
PAT‐ Okay so that's what they call, can we mark this down?
PAT 2‐ Did you say its three?
DOC‐ Plus three, Gleason six,
PAT‐ There's places on that book they gave is very helpful so
DOC‐ Sure, sure
PAT‐ I want to be able to fill that out, uh, as we're going around. We're going to have some questions for you.
**Request for information or question**
PAT‐ Now, again I'm going to ask the questions Doc, what grade is it?
DOC‐ Low grade
PAT‐ Low grade, and it's, the cells are they in the stage….. alright, here's what, and I don't know I'm not a doctor, my son is but I'm not. In this booklet that they got here, here we go. I'm a stage one or two right?
DOC‐ Stage one.
**Uses booklet to question doctor**
DOC 1‐ That's low grade, it goes from the Gleason.
PAT‐ If the high is ten, you're over half.
DOC 2‐ Right, but the lowest grade that they call is six
DOC 1‐ Right
DOC 2‐ It's a scale of six to ten, not, not zero to ten
PAT‐ Not according to this book
DOC 2‐ Yeah. Well the pathologists don't call Gleason fives anymore, they used to, but they don't anymore.
**Expression of concern**
*Example 1*
PAT‐ Whenever I start getting upset or nervous about this I can take this out and start reading through it again. The way things are explained in here kind of calms you down.
DOC‐ Well yeah, that's good to know.
*Example 2*
DOC‐ Do you have access to the web?
DOC‐ Is that the material that they give you?
PAT 2‐ It's the book, yeah. We both read it so
DOC‐ I see
PAT 1‐ Gives you something
DOC‐ It's good, no, it's an excellent resource. You know,
PAT 1‐ Well, it makes you sweat.

Occasionally, patients referred to the booklet to explain how worried they were about their prostate cancer or specific treatments. The DA, in these instances, appeared to serve as either reassurance, or as a vehicle for expressing concern to the physician. (See Table 5.) Patient references to the DA to challenge the physician's recommendation were rare.

### 3.2 Predictors of booklet talk

Results of the first mixed effects regression model revealed that only education and time in the clinical encounter predicted reference to the booklet (ie, booklet talk). Specifically, higher education was associated with lower odds of the booklet's being discussed in the clinical encounter. Odds of booklet talk among patients with some college or trade school education had an OR = 0.45 (95% CI = 0.22‐0.90, *P* = .024) compared with those with a high school degree or less. Odds of booklet talk of those with a 4‐year college degree and beyond had an OR = 0.42 (95% CI = 0.18‐0.96, *P* = .041) compared with those with a high school degree or less. Longer time spent in the clinical encounter predicted higher odds of the DA being discussed (OR = 1.03, 95% CI = 1.00‐1.06, *P* = .034). We have previously shown that time in the encounter varied widely and was modestly associated (*r* = .24, *P* = .01) with the IDM score. In this analysis, time in the encounter, but not IDM score predicted booklet talk. Patient race, DA type and time spent reading the DA were not significant predictors of booklet talk.

In the second model, we tested whether adding physician level variables capturing physician experience and their quality of informing patients about treatment decision‐making would cause patient education and time in the encounter to drop out as significant predictors. Patient's level of education remained significant, but only for those with some college or trade school education (OR = 0.46, 95% CI = 0.23‐0.91, *P* = .027). In both models, men with only a high school education referred to the booklet more often than more highly educated men. Time in the clinical encounter also remained significant. In longer clinical encounters, the booklet had higher odds of being discussed (OR = 1.03, 95% CI = 1.00‐1.07, *P* = .034). Patient race, DA type, time spent reading the DA, the IDM score and physician age were not significant predictors of booklet talk.

## 4 DISCUSSION

While many DAs have been shown to be effective in translating medical evidence for patients, they are not routinely used in practice.^1^, ^3^ Our results contribute to better understanding of this implementation conundrum. We found that DAs appeared to function not to open up deliberations about how to balance benefits and harms of competing treatments, but to allow patients to ask narrow technical questions about recommended treatments. This was contrary to expectations, as we chose high‐quality DAs, shown previously to be engaging to patients.^19^ We found no evidence that DAs functioned to facilitate shared decision‐making in the encounter.

Direct references to DAs occurred in over half of encounters. Direct references to the DA were more frequently initiated by patients than physicians. This may in part, be attributable to the fact that physicians did not receive any training in DA use, while patients were asked to use the DAs to prepare for the encounter. However, the analysis of function codes revealed that patients largely used the DA to corroborate what the physician said or to request more technical detail. Specifically, 41% of booklet talk functioned to validate or prompt additional discussion of a topic (“learn more”); the most common topic discussed when referencing the DA was details of specific treatment options. Patients only used the DA as a platform for asking the physician a question in 12% of transcripts (see Table 2). Patient questions were often about prognosis and treatment options. While it may be that patients with specific questions brought the DA along, it suggests that encouragement to patients to bring a DA to the encounter may increase the likelihood that the DA content is discussed in the encounter.

Patients did not use the DA to articulate their outcome preferences and goals as encouraged in the DAs themselves. Nor did they use the DA to bring up treatment outcomes or use the DA to say what side‐effects concerned them. Rather, booklet talk fit into a physician‐driven medical routine focused on understanding biopsy results and reviewing treatment options to settle on a treatment. This use of the DA is consistent with a companion analysis of this data set showing that physician recommendations dominated treatment preference.^41^ That analysis showed that for these low and intermediate risk prostate cancer patients, treatment decisions were based largely upon urologists’ recommendations, and not on patients’ personal views of the relative pros and cons of treatment alternatives. Urologists’ recommendations, in turn, were influenced heavily by medical factors (age and Gleason score) but were unrelated to patient preferences. While the presence of a DA did not appear to influence the informing and treatment choice process, it did appear to support patient understanding of treatment choices. In addition, both DA booklets stimulated booklet talk, suggesting that actual use of the DA in the encounter is a generalizable phenomenon across different DAs.

Regression analysis showed that less well‐educated patients were more likely to mention the DA. This finding is not unexpected. In our prior research, we found that patients with less education gained more knowledge from a DA.^42^ It is one of the unique characteristics of DAs that those who are less knowledgeable before reading a DA gain the most knowledge. DAs are also designed to provide an authoritative source to help patients ask questions.^1^ Time in the encounter, but not the quality of physician informing (IDM score) predicted booklet talk. This lends support to earlier findings that discussing patient questions raised by a DA may take a small amount of extra time in the encounter. The Cochrane Review of DAs shows that the association of DA use with time in the encounter is highly variable, sometimes associated with shorter and sometimes with longer encounters (range −4 minutes to +23 minutes), with an average of 2.6 minutes longer^1^. It is important to note that in this study, which over‐sampled African American patients, race was not a predictor of booklet talk. This suggests that minority and white patients were equally likely to use the DA in the encounter.

While there are studies of patient‐clinician communication focused on measuring the presence of shared decision‐making,^43^ we know of none that investigates how DAs function in real time during the consultation. We extend previous research on DA use by describing what issues from the DA were brought up, and how they functioned in doctor‐patient communication about treatment decisions for localized prostate cancer treatment. This augments prior research about DA effects based on self‐report measures of patient‐clinician communication. In these geographically distributed Veterans Administration clinics, DAs were used as an adjunct to physician treatment recommendations. A limitation of this study is that only explicit mentions of DAs were coded. Other patient questions may have been stimulated by DAs that were not explicitly linked to the DA as booklet talk. However, as the field moves towards DA use to support decision‐making that reflects patient values, it will be critical to understand what actually happens during DA implementation in clinical encounters across settings.
