# Supplementary material for: Decision aid use during post‐biopsy consultations for localized prostate cancer
Source: Health Expect. 2017 Sep 7;21(1):279–87. doi: 10.1111/hex.12613 (PMC5750733; doi:10.1111/hex.12613)
Supplement: Supplementary file 1 [file HEX-21-279-s001.doc]

Online Appendix

Decision Aids: The two DAs were chosen for the parent study to address the problem that interventions using decision aids (DA) increase knowledge, but rarely affect patients’ treatment choice. The central hypothesis was that high literacy demands in most DAs and the absence of explicit support for shared decision making might explain the lack of impact on choice. The plain language DA was developed by the Michigan Cancer Consortium (MCC) to use plain language and to adhere to the standards of the International Patient Decision Aids Consortium (IPDAS). The comparison standard language DA was developed by the National Comprehensive Cancer Network and the American Cancer Society(NCCN) and was chosen because of its high-quality information and the high credibility of the sponsoring organizations. Both decision aids used the terminology “watchful waiting” because active surveillance was not a commonly used term when this study. Therefore, we use watchful waiting throughout.

The Plain Language DA**,** “Making the Choice: Deciding What to Do About Early Stage Prostate Cancer” was developed by the Michigan Cancer Consortium (MCC), was designed for patients making treatment decisions about clinically localized prostate cancer and did not include information about advanced or metastatic prostate cancer. This DA incorporated text and document layout features that support readers in understanding basic health information needed to make health decisions. Patient testimonials and language throughout the decision aid conveyed the message that each man should make the decision that was right for him. It was designed to be consistent with the IPDAS guidelines, comparing the probabilities of benefits and harms (side effects of treatments), using both text and numbers of people out of 100. The current version of the MCC DA can be found at [www.prostatecancerdecision.org](http://www.prostatecancerdecision.org/).

The Standard Language DA*,* “Prostate Cancer: Treatment Guidelines for Patients”, was developed by the National Comprehensive Cancer Network (NCCN) to translate treatment guidelines into a patient-friendly format. It was aimed at patients with either early or later stage prostate cancer. The information in the DA was based on the NCCN’s Clinical Practice Guidelines for patients. It included common side effect rates in percentages, and used decision trees to present treatment options. The DA was the first by national charities in the US to present treatment and side effects in detail and explains that localized prostate cancer is a chronic disease that is unlikely to cause death. Options were framed as choices men can make with guidance from their doctors and the NCCN guidelines. The current version of the NCCN DA can be found at <https://www.nccn.org/patients/guidelines/prostate>**.**

Quality Comparison of DAs An assessment of the quality of the two DAs was obtained by having independent raters at a different institution use the IPDASi scoring system, a validated short version of the original IPDAS quality criteria. Both DAs were shown to be of high quality. The MCC document provided data in more quality domains than did the NCCN document. Since we did not have data about the development of the NCCN document, the summary scores cannot be validly compared. Inspection of the domain scores where there was complete data shows that the MCC DA scored higher on the domains of Information Relevance, Evidence, Probabilities and Guidance. The two DAs showed similar scores on Disclosure of Conflicts of Interest, and on Values.
